# The beneficial effects of cognitive training with simple calculation and reading aloud in an elderly postsurgical population: study protocol for a randomized controlled trial

**DOI:** 10.1186/s13063-016-1476-0

**Published:** 2016-07-22

**Authors:** Kay Kulason, Rui Nouchi, Yasushi Hoshikawa, Masafumi Noda, Yoshinori Okada, Ryuta Kawashima

**Affiliations:** 1 Department of Functional Brain Imaging, Institute of Development, Aging and Cancer (IDAC), Tohoku University, Sendai, 980-8575 Japan; 2 Creative Interdisciplinary Research Division, Frontier Research Institute for Interdisciplinary Science (FRIS), Tohoku University, 4-1 Seiryocho, Aobaku, Sendai, 980-8578 Japan; 3 Smart Aging International Research Center, Institute of Development, Aging and Cancer (IDAC), Tohoku University, Sendai, 980-8575 Japan; 4 Human and Social Response Research Division, International Research Institute of Disaster Science (IRIDeS), Tohoku University, Sendai, 980-0845 Japan; 5 Department of Thoracic Surgery, Institute of Development, Aging and Cancer (IDAC), Tohoku University, Sendai, 980-8575 Japan; 6 Division of Developmental Cognitive Neuroscience, Institute of Development, Ageing and Cancer, Tohoku University, Sendai, 980-8575 Japan

## Abstract

**Background:**

This project proposes a pilot study to investigate the positive healing effects of cognitive training with simple arithmetic and reading aloud on elderly postsurgical patients. Elderly patients undergoing surgery have an increased risk of Postoperative Cognitive Decline (POCD), a condition in which learning, memory, and processing speed is greatly reduced after surgery. Since elderly patients are more likely to exhibit symptoms of POCD, the incidence is increasing as the population receiving surgery has aged. Little effort has been expended, however, to find treatments for POCD. Learning therapy, which consists of a combination of reading aloud and solving simple arithmetic problems, was developed in Japan as a treatment for Alzheimer’s Disease to improve cognitive functions. Because patients with Alzheimer’s Disease experience similar issues as those with POCD in learning, memory, and processing speed, a cognitive intervention based on the learning-therapy treatments used for Alzheimer’s Disease could show advantageous outcomes for those at risk of POCD.

**Methods/design:**

Cognitive function will be measured before and after surgery using three different tests (Mini-Mental Status Exam, Frontal Assessment Battery, and Cogstate computerized tests). Subjects will be randomly divided into two groups—one that receives a Simple Calculation and Reading Aloud intervention (SCRA) and a waitlisted control group that does not receive SCRA. To measure cognition before and after the intervention, the previously mentioned three tests will be used. The obtained data will be analyzed using statistical tests such as ANCOVA to indicate whether the cognitive intervention group has made improvements in their cognitive functions. In addition, questionnaires will also be administered to collect data on mental and emotional statuses.

**Discussion:**

This report will be the first pilot study to investigate the beneficial effects of SCRA on elderly surgical patients. Previous studies have shown sufficient evidence on the effectiveness of learning therapy in healthy elderly people and in those with Dementia. Therefore, this study will clarify whether SCRA can improve cognitive function in the more specialized group of elderly surgical patients.

**Trial registration:**

University Hospital Medical Information Network Clinical Trial Registry, UMIN000019832. Registered on 18 November 2015.

**Electronic supplementary material:**

The online version of this article (doi:10.1186/s13063-016-1476-0) contains supplementary material, which is available to authorized users.

## Background

Memory loss and lack of concentration are symptoms that often occur in patients who have undergone surgery. These symptoms are part of a condition called postoperative cognitive decline (POCD), which is not an official diagnosis. Changes in cognitive abilities have been reported in elderly patients since the 1950s, and anesthesia has often been thought to be a potential culprit [1]. In 1955, Bedford [1] published a retrospective review of 1193 elderly patients who had surgery under general anesthesia over a 5-year period. He concluded that the cognitive problems were related to anesthetic agents and hypotension and that “operations on elderly people should be confined to unequivocally necessary cases.”

While most patients exhibiting POCD naturally recover from the condition in the 6 months after surgery, nearly 2 % of POCD cases last until death [1]. Thus, POCD can be divided into three categories: acute, intermediate, and long-term. Acute POCD is often used to describe cognitive decline detectable in the 1 week after surgery, intermediate POCD refers to cognitive decline detectable in the 3 months after surgery, and long-term POCD is used to describe changes lasting 1–2 years following surgery. A decline in cognition leads to difficulty in performing basic everyday activities [2–4], which results in a loss of independence [5]. POCD is also strongly associated with premature departure from the labor market [6]. Furthermore, people with POCD are at higher risk of death in the first year after surgery [7]. Consequently, maintaining or improving cognitive function in older adults is drawing increasing attention [8–23].

An international multicenter study on POCD reported memory impairments in 26 % of patients 60 years and older, with deficits reported to last for months to years [24]. Most notable instances of POCD are observed after cardiac and thoracic surgery [25]. Age is nevertheless the biggest risk factor for POCD [5, 24]. In addition, even healthy elderly adults may experience a decline in several cognitive functions, including memory [26], attention [27], executive functions [28, 29], and processing speed [30]. POCD is especially a problem because over the past 20 years, the number of older people undergoing surgical procedures has increased faster than the population is aging [25, 31].

However, very few studies have been conducted on the different methods for enhancing cognitive functions in a postsurgery elderly population. Nevertheless, several cognitive training programs have been shown to improve cognitive functions, including memory [16, 32], processing speed [19, 33, 34], executive function [17, 35], and attention [36] in a healthy elderly population. One such cognitive training method, learning therapy, has several beneficial points, which make it more appealing over other methods: 1) it can be conducted in healthy elderly people and in those suffering from dementia, 2) it is cost-efficient because special machinery and devices are unnecessary, and 3) the burden on participants is minimal because learning therapy takes only 15–30 minutes each day.

Learning therapy consists of two simple and easy training tasks (reading Japanese aloud and solving easy arithmetic) derived from knowledge of neuroscience. Brain imaging studies indicate that reading sentences or words aloud [37–41] and simple arithmetic operations [42–44] activate the frontal cortex and of the temporal and parietal association cortices. Thus, learning therapy was specially designed to stimulate these cortices via the cognitive tasks, thereby promoting improvement in the function of these cortices [45, 46]. Previous studies using learning therapy have demonstrated that it can improve executive functions and processing speed in healthy elderly people. One such study by Uchida and Kawashima [35] conducted a randomized controlled trial using learning therapy for healthy elderly people. Participants were divided into a learning therapy group and a waitlisted control group. The learning therapy group was required to do two training tasks for 5 days a week: reading Japanese aloud and conducting simple calculations. After 6 months, the learning therapy group showed improved scores in the frontal assessment battery (FAB), which measures executive function [47–49], and a digit-symbol substitution test, which measures processing speed [50]. These results suggest that learning therapy beneficially affects some cognitive functions in elderly people.

However, learning therapy is conducted every day with a weekly visit to the learning facility. Since the participants in this pilot study will be undergoing surgery, such an intense course of training is not reasonable. As a result, this pilot study will incorporate a modified, less-intensive version of learning therapy, and henceforth will be referred to as the simple calculation and reading aloud (SCRA) intervention. To measure changes in cognitive function due to surgery, this study will incorporate several cognitive tests, in addition to the conventionally used MMSE-J. These tests will determine what cognitive functions decline, and to what degree. Furthermore, we will examine to what extent the cognitive intervention improves cognitive functions in the elderly population after surgery. Several health questionnaires will also be administered to determine mental and emotional wellbeing. In addition, the type of general anesthetic administered, the amount of anesthetic administered, the anesthesia duration, the surgery duration, age, and sex will be noted.

### Purpose

This study is an open-label randomized control trial utilizing a between-group design to examine 1) to what extent cognitive function is reduced after surgery and 2) whether cognitive training focusing on SCRA will improve cognitive functions in elderly patients after surgery. The first and second aims of this study are independent of each other. A decline in SCRA function after surgery is expected, especially in the elderly population from which the participants will be chosen. Previous results conducted in a healthy elderly population suggest that cognitive intervention should improve cognitive functions. If the cognitive intervention is successful for treating patients with higher risk of POCD, this noninvasive method could greatly improve their quality of life.

As mentioned above, no standardized method exists to diagnose a patient with POCD. In addition, due to the limited number of participants, we lack the data necessary to mirror previously employed methods [5, 24, 25] that determine whether each individual patient has POCD. Therefore, this study will investigate 1) the effect surgery has on the cognitive functions of the group of participants undergoing surgery and 2) the effects of SCRA intervention on the same participants, all of whom will fall under the population category of “elderly” and “post-surgical.”

## Methods/design

The trial protocol was developed according to the SPIRIT and CONSORT guidelines for randomized controlled trials [51]. Please see Additional file 1 (CONSORT guidelines) and Additional file 2 (SPIRIT).

### Randomized controlled trial design and setting

This study is an open-label, randomized, controlled trial conducted in Sendai City, Miyagi Prefecture, Japan. Each participant in this study will provide written informed consent prior to participating. The protocol of the study and the consent form have been approved by the Ethics Committee of the Tohoku University Graduate School of Medicine. This study was registered with the University Hospital Medical Information Network (UMIN) Clinical Trial Registry (UMIN000019832).

**Fig. 1:**
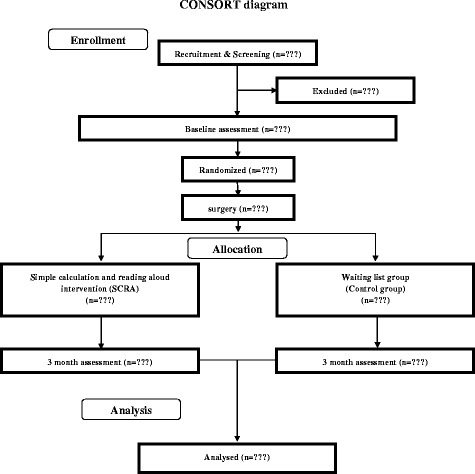
CONSORT flowchart

**Fig. 2:**
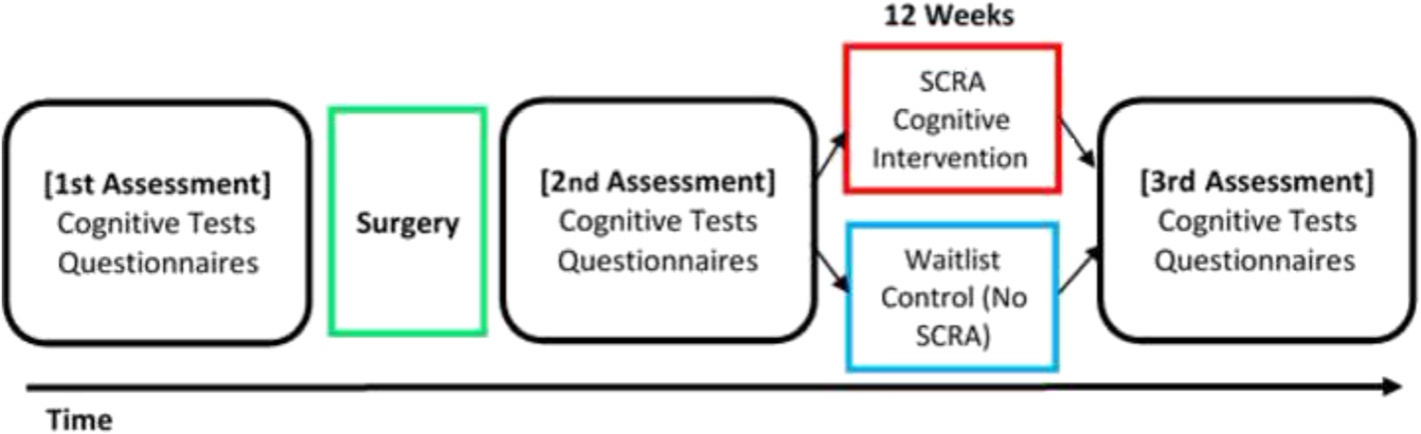
The timeline of the study indicates when the questionnaire and cognitive function tests will be administered. The tests will be administered to the participant on three occasions. The first time will be before the surgery as a baseline. The tests will be administered a second time after surgery to determine whether the surgery affected cognitive functions. The participants will be randomly split into two groups: 1) the group that receives SCRA cognitive intervention and 2) the waitlist control group. The cognitive intervention group will continue the cognitive intervention for 12 weeks. The no-SCRA control group will continue life as normal for 12 weeks. After 12 weeks, both groups will be administered the final round of tests to determine if SCRA intervention improved cognitive functions. The tests will be conducted in a ward or outpatient room at Tohoku University Hospital of Thoracic Surgery

To assess the effects of SCRA on elderly patients who have undergone surgery, all the participants will be randomly assigned to one of two groups: the SCRA intervention group and the waitlisted control group, which does not undergo the SCRA intervention. The study design is shown in Figs. 1 and 2.

### Recruitment of participants

Respiratory patients undergoing lung surgery will be recruited from Tohoku University Hospital. Patients admitted to the hospital prior to surgery will be informed about the study. If interested in participating, the patient will receive both a written and a verbal explanation of the study and be asked to sign the approved informed consent form.

### Eligibility criteria

Approximately 50 volunteers will be recruited from respiratory patients undergoing lung surgery with general anesthesia lasting 2–8 hours. As previously mentioned, this is a pilot study, so 25 volunteers in each group should be enough to provide information on the changes in cognitive function. Participants must be native Japanese speakers who are 60 years and older and self-report to be right-handed. Participants should be unconcerned with their memory functions. A thoracic surgery physician from the Tohoku University Hospital will refer potential participants. Other data that will be recorded includes the type of general anesthesia, the amount of anesthesia administered, duration of anesthetic administration, duration of surgery, age, and sex. These additional data will be collected by the collaborating surgeons.

### Randomization and blinding

Participants will be randomly placed into one of two groups: 1) the group undergoing SCRA cognitive intervention and 2) the no-SCRA waitlisted control group. Random assignment using an online computer program (http://www.graphpad.com/quickcalcs/index.cfm) will take place after informed consent and baseline testing. Participants are stratified by gender and randomized to SCRA or waitlisted control groups. In order to obtain a similar size for both groups, blocked randomization (block size 4) is applied with an allocation ratio of 1:1. Since the participants of the study are patients undergoing cognitive intervention or are being waitlisted, designing a blinded study is difficult. As a result, the trial design is open-label.

### Determining postsurgical cognitive function

First, the Mini-Mental State Examination-Japanese (MMSE-J) will be administered to the participant after receiving consent. The MMSE-J is a 30-point questionnaire used extensively in both clinical and research settings to measure cognitive impairment [52]. To assess dementia, clinicians will consider a person’s MMSE-J score along with their history, a physical health exam, symptoms, and results from other tests. A higher MMSE-J score indicates better cognitive performance. The MMSE is the most common mental status test used to determine POCD [53, 54]. For test-retest intervals of 2 months or less, the MMSE has been shown to have good test-retest reliability [53, 55, 56].

Following the MMSE-J, the participant will be given the Frontal Assessment Battery (FAB). The FAB is an 18-point questionnaire that is commonly performed at bedside or in a clinical setting to help measure cognitive impairment in executive functions [47–49]. Again, higher scores indicate better performance. The Japanese translation of the FAB has previously been shown to be comparable to the original English versions and has a good test-retest reliability at 3 weeks [48, 49]. Both the MMSE and FAB are commonly administered in studies determining cognitive decline [57–59].

After the FAB, the participant will be given a laptop running the Cogstate software to measure the speed of processing, visual attention, and visual learning and memory via Cogstate’s “Brief Battery” (https://cogstate.com/). This computerized battery has been shown to effectively determine cognitive decline [25, 60]. Previous studies on the test-retest reliability of the CBB have indicated that there are no retest-related increases in scores after 1 month. However, the retest scores have been shown to increase at 1 week or less [60–62]. Additionally, the Japanese version of the CBB has previously been shown to yield reliable results for Japanese subjects [63]. This computerized test will be administered via a laptop. Trained project investigators will give detailed instructions and will be nearby to provide reassurance to the elderly participants and to help with any confusion regarding computer use. In order to measure these indicators, the software will run four tasks in sequence, which lasts approximately 15 min in total. First, the software will run a detection task to measure the speed of processing. The participant will be asked to hit the “Yes” button when the trump card turns face up. The unit of measurement will be Log10 milliseconds of the reaction times for correct responses. Incidentally, a lower score indicates better performance. The next task will be an identification task to measure visual attention. The participant will be asked to press “Yes” if the card is red and “No” if the card is black. Again, the unit of measurement is Log10 milliseconds of the reaction times for correct responses. The third task is a one-card memory task in which the participant is asked to press “Yes” if they have already been shown the card. The accuracy of performance will be measured with the arcsine transformation of the square root of correct responses. Therefore, a higher score indicates better performance. The final task is a working memory task called “One Back,” in which the participant is asked to press “Yes” if the card is the same as the previous card, and “No” if it is different. The accuracy of performance is measured using the arcsine transformation of the square root of the proportion of correct answers. Again, a higher score reflects better performance.

These tests will be administered and scored by trained project investigators, and they will be administered to the participant three times: 1) prior to surgery as a baseline, 2) after the surgery to determine a decline in cognition (about 1 week after surgery), and 3) 12 weeks after surgery to determine the effects of SCRA. The type of general anesthesia administered, the amount of anesthesia administered, the anesthesia duration, the surgery duration, age, and sex will be noted. Additionally, several health questionnaires will be administered to determine emotional and mental wellbeing.

### Cognitive intervention group

**Fig. 3:**
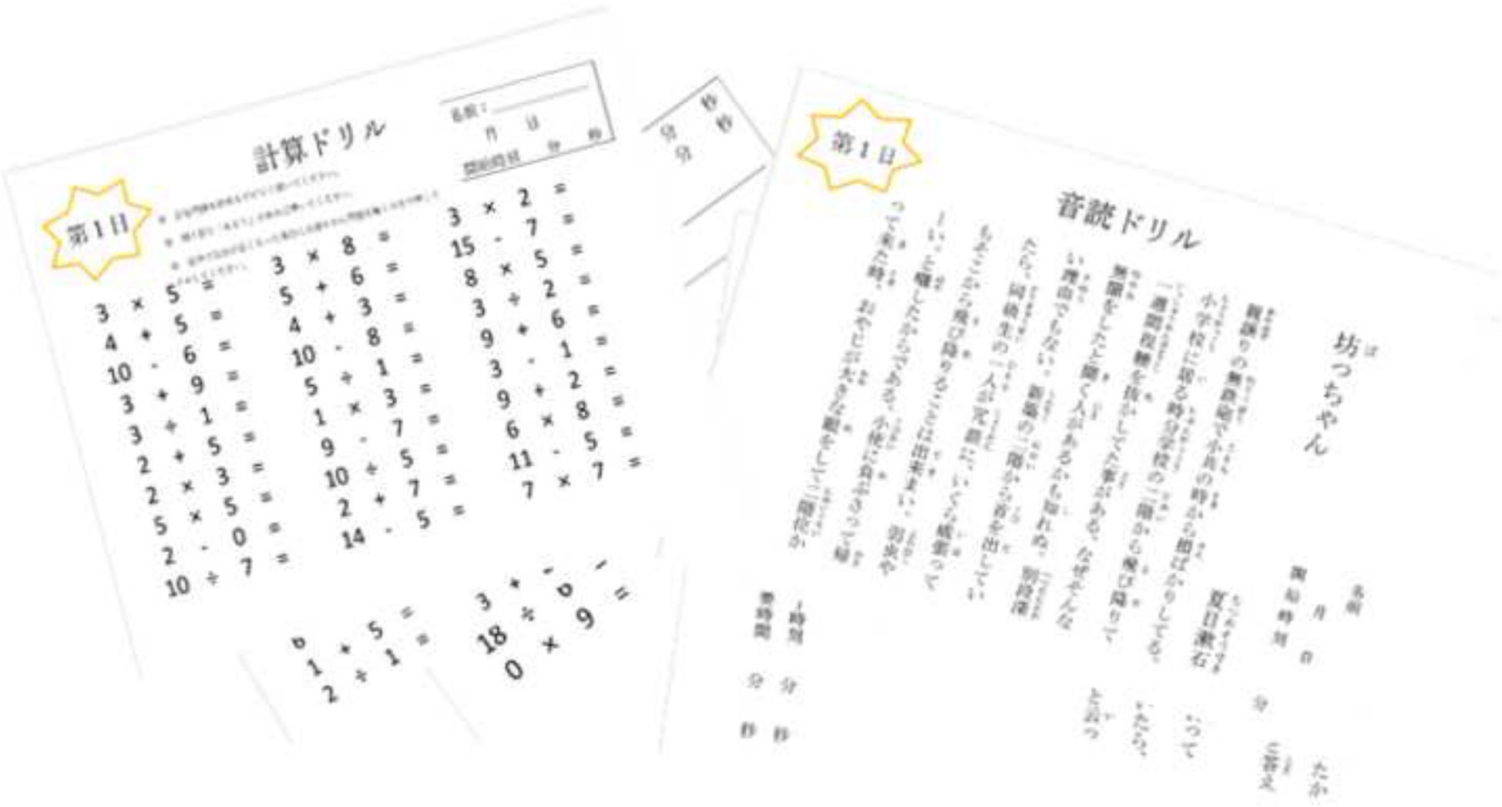
Sample Simple Calculation and Reading Aloud (SCRA) cognitive intervention worksheets that will be given to the participants randomly assigned to the experimental group. Reading aloud (*right*) and simple arithmetic (*left*)

The group receiving the SCRA cognitive intervention will be randomly selected from the entire pool of participants, without regard to whether there was a decline in cognitive functions after surgery. SCRA intervention will be conducted at home 3–5 times a week for 30 minutes. The cognitive intervention method consists of two simple tasks: 1) solving simple arithmetic, and 2) reading aloud in Japanese. Various materials will be prepared from Dr. Ryuta Kawashima’s published book series “Training the Brain: The Adult’s Arithmetic Drills” 「脳を鍛える大人の計算ドリル」and “Training the Brain: The Adult’s Verbal Reading Drills” 「脳を鍛える大人の音読ドリル」. These training materials have previously been shown to be effective [35, 45]. The difficulty of arithmetic materials used will range from solving single-digit addition to double-digit division. Materials for reading Japanese aloud consist of 1–2 page stories and essays. Example training worksheets are shown in Fig. 3. The training materials will be given to the participant during their scheduled hospital visits (at discharge, 2 weeks postsurgery, 1 month post-surgery, and 2 months postsurgery). The percentage of correct answers and the time it takes to complete each task will be recorded.

Additionally, during the scheduled hospital visits, we will also ask about daily habits. Knowledge of the individual differences in emotional state is important because it could possibly affect the study results.

This cognitive intervention will continue for 12 weeks. Participants will be given all the tests mentioned above (MMSE-J, FAB, CBB, and health questionnaires) one final time at the end of the 12 weeks.

### Waitlisted control group

The waitlisted control group will also be randomly selected from the pool of participants after surgery. During the duration of the study, the waitlisted control group will continue life as usual and will not receive SCRA cognitive intervention. After completing the study, they will be given the opportunity to experience the cognitive intervention. No placebo will be used for this group. Results from previous intervention studies [32, 64] report that a placebo group is unnecessary for this type of study because no differences were observed in cognitive function improvement between the placebo group and the control groups. These participants will also travel to the hospital to meet with their doctors. As with the experimental group, during these visits, control group participants will also be queried about their daily habits. Twelve weeks after surgery the control group will be administered the final round of tests (MMSE-J, FAB, CBB, and health questionnaires).

### Outcomes

The outcome measures for all participants are the test scores from the MMSE-J, FAB, computerized tests, and health questionnaires. Additionally, the type of general anesthetic administered, the amount of anesthetic administered, the anesthesia duration, the surgery duration, age, and sex will be recorded.

### Sample size

This is a pilot study. Because a similar study has not been conducted, calculation of a sample size is not possible. However, we will aim to include 50 participants.

### Data management

Personal information and data for all experiments are handled by the personal information manager director of Tohoku University. Access to information requires a key that is securely kept and has limited access. These internal checks and balances ensure the security of all data and personal information. After the end of the experimental period, the data will be consolidated, and any information linking data back to the participant will be discarded to ensure that the data is truly anonymous. Data destruction will not be conducted.

### Statistical analysis

This study is designed to 1) determine whether there is a decline in cognition after lung surgery in elderly patients and 2) evaluate the beneficial effect of SCRA cognitive intervention in this patient population. The analysis will be based on the intention-to-treat principle.

For comparison of cognitive function before and after surgery, a Wilcoxon Signed-Rank test will be used to compare all participants in both groups. This will determine whether cognitive functions declined postsurgery. Participants will have been randomly placed into a group receiving SCRA intervention, or a waitlisted control group. To evaluate the effects of the cognitive intervention, we will calculate the change in score (post-training score minus pre-training score) for each measure and conduct a between-group variance ANCOVA test. The change in scores is the dependent variable, while the treatment group (cognitive intervention group, waitlisted control group) is the independent variable. Pretraining scores in the dependent variable, sex, and age categories are the covariates to exclude the possibility that any pre-existing difference of measure between groups will affect the result of each measure and to adjust for background characteristics. This is a pilot study that will likely be conducted with a small number of participants, and the effects are unlikely to be significant.

The level of significance is set at *p* < 0.05. However, since this study includes six different cognitive tests (MMSE-J, FAB, and four computerized tests), we will use Benjamini and Hochverg’s false discovery rate (FDR) correction methods to adjust the *p* values [65]. As a result, the statistical threshold of the behavioral data was set at *p* < 0.05, FDR-corrected. Moreover, we will report eta squared (h2) as an index of the effect size. This index is the standardized difference in the change score between the intervention groups (cognitive intervention group, waitlisted control group). In actuality, h2 ≥ 0.01 is regarded as a small effect, h2 ≥ .006 as a medium effect, and h2 ≥ 0.14 as a large effect [66]. The level of significance is set at *p* < 0.05. Missing data are imputed using the multiple imputation method, as implemented in the Statistical Package for the Social Sciences (SPSS). SPSS uses a fully conditional specification, or chained equations, imputation algorithm. Essentially, incomplete variables are individually imputed and uses the filled-in variable as a predictor in all subsequent steps. Linear regression is used for continuous variables, and logistic regression is used for categorical variables [67]. All randomized participants are included in the analyses in line with their allocation, irrespective of how many sessions they complete (intention-to-treat principle). All statistical analysis will be conducted using SPSS software (Statistical Package for Social Science).

### Data monitoring and auditing

We will follow the recommended guidelines for clinical research in Japan (http://www.mhlw.go.jp/topics/bukyoku/seisaku/kojin/dl/161228rinsyou.pdf). According to the guidelines, data monitoring by a third party is unnecessary for this study since we are not providing participants with any medications, nor are we conducting surgery.

### Risks and benefits to participants

Participants are unlikely to encounter any serious risks or burdens. Nevertheless, the three cognitive tests together are expected to take 1 hour. Therefore, participants will possibly experience fatigue and discomfort during testing. The participant will be informed in advance that, should they feel any discomfort, the test can be interrupted at any time. In addition, this study requires participants to undergo cognitive training for 12 weeks. The training tasks (reading aloud and solving simple arithmetic) are not particularly difficult and should not cause the participants any pain. However, if for some reason the participant experiences pain, they will have been previously informed that they may immediately interrupt the training.

In accordance with the provisions of the university, the participants will be given a monetary reward. The reward amount is based on the number of hours invested. Therefore, should the participant decide to leave partway through the study, the reward amount received will coincide with the number of hours they partook.

### Ethics and dissemination

The protocol of the study and the consent form were approved by the Ethics Committee of Tohoku University Graduate School of Medicine. Participants will be given both an oral explanation and a written file that documents the details of the study. Upon understanding, the participant will be asked to give written consent by signing the consent form approved by the ethics committee. Participants may choose to withdraw from the study at any point in time.

Should they choose to do so, their data will be destroyed.

All participants will be given subject identification numbers to which all the data will be tied. The file records that connect each participant to their identification number will be securely kept on lab servers during the duration of the study. After the study is concluded, the file will be destroyed.

This study is carried out using the operating grants of the group of Tohoku University of implementation officer. For the practice of the present research program, no relationship exists between the particular companies. In other words, no conflict of interest exists in this research project.

As previously mentioned, all personal information and data for the experiment are handled by the personal information manager director of Tohoku University. Access to information requires a securely kept key with limited access. These internal checks and balances ensure the security of all data and personal information.

### Dissemination policy

Results of the study will be formally published in a journal. Participants, healthcare professionals, the public, and other relevant groups will not be individually notified of the results. There are no publication restrictions to note. Authorship of the study belongs to those specifically listed; no professional writers were involved. Currently, no plans exist to grant public access to the full protocol, participant-level dataset, and statistical code.

## Discussion

This pilot study is designed to investigate the beneficial effects of SCRA intervention in elderly surgical patients. As previously mentioned, age has been indicated as a major risk factor for postoperative cognitive decline and is found in approximately 26 % of individuals over 60 years old [24]. This is the first study to investigate improving cognition after surgery using a noninvasive intervention in an elderly patient population. Therefore, this present study will clarify whether the SCRA intervention is beneficial to cognitive functioning in the elderly surgical population.

In our study, we will use simple paper and pencil training tasks (reading aloud and simple arithmetic). Many tasks in previously conducted studies were complex tasks using computers [16, 36, 68–70]. While computerized tasks allow data to be easily and precisely recorded, the use of computers is unfamiliar to the elderly population [71–74]. Using computers could cause frustration, negative emotions, and decreased motivation towards the task. The cognitive intervention tasks, on the other hand, are more familiar to the elderly population and, therefore, are expected to encourage the willingness of the elderly to participate [75]. Again, the SCRA cognitive intervention that will be used in this study is a cognitive training program focused on reading aloud and simple arithmetic and has been shown to improve executive functions and processing speed in both healthy elderly people and those suffering from dementia [35, 45].

This study does include several limitations. First, the intervention period is 3 months. Previous studies have shown that short-term interventions (i.e., 4–6 weeks) are sufficient to improve cognitive functions in elderly people [14, 16, 76, 77]. A shorter intervention would reduce the cost for elderly people seeking cognitive intervention, and therefore, a shorter study may also be beneficial. In addition, controlling participant variables (i.e., excluding those with severe hypertension, Parkinson’s disease, multiple sclerosis, thyroid disease, stroke, heart disease, and diabetes and those using medications that affect cognitive function, etc.) was not realistic due to the short recruitment period. In addition, participation was limited to patients receiving lung surgery under general anesthesia for 2–8 h, and therefore, the results will not be applicable to all types of surgery. Therefore, the data collected are unlikely to reveal significant results. The study should be repeated with large sample sizes and in patients undergoing different types of surgery with a range of duration of anesthesia and variety of anesthetics.

In addition, the waitlisted control group was not given a placebo. This is because it was deemed unreasonable to make elderly patients recovering from surgery complete tasks that would not have any effects. The lack of a task for the control group may introduce a bias because the waitlisted participants may realize that they are expected to perform worse than their experimental counterpart, and vice versa. This expectation may lead to differences in motivation for completing the various measures. Nevertheless, as previously mentioned, several studies have indicated that a placebo is not necessary for this type of study [32, 64]. The purpose of this study is to determine the effects of SCRA in the elderly population after surgery. The waitlisted control group is sufficient for this study since they represent the general elderly population after surgery.

Another limitation to consider is the number of participants. Because the recruitment period is short, the number of participants is severely limited. However, this is a pilot study intended to explore the possible effects of cognitive intervention in the elderly population after surgery, and therefore, the small sample size should suffice to provide the basic groundwork for future investigations.

In summary, this study is the first to explore the beneficial effects of SCRA cognitive intervention in unhealthy elderly adults undergoing surgery. This study is designed to provide preliminary support for the effectiveness of the cognitive intervention as a means to treat postoperative cognitive decline. While cognitive functions can be affected by surgery [24], most cognitive functions also decrease with age [23]. Given that these functions are strongly correlated with daily activities [2, 3, 22], our results can reveal the effects of SCRA intervention for the elderly post-surgical population.

The results of this study will potentially have important implications for treating cognitive decline in the elderly population after surgery. As people live longer, the elderly population is increasing. In Japan, 25 % of the population is older than 65 years of age. This is the highest proportion in the world and represents a 3 % increase between 2009 and 2013 (World Bank, 2014). The increase is also reflected in the surgical population. In fact, over the past 20 years, the number of older people undergoing surgical procedures has increased faster than the population is aging [31]. As a result, the results of this pilot study potentially have wide applicability.

## Trial status

Recruitment of participants started in January 2016 and will end in August 2016.

## Abbreviations

ANCOVA, analysis of covariance; CONSORT, Consolidated Standards of Reporting Trials; FAB, Frontal Assessment Battery; MMSE, Mini-Mental Status Exam; POCD, postoperative cognitive decline; RCT, randomized controlled trial; SCRA, simple calculation and reading aloud; SPSS, Statistical Package for the Social Sciences; UMIN, University Hospital Medical Information Network

## Additional files

Additional file 1: CONSORT checklist. A table specifying where any CONSORT item has been addressed in the protocol manuscript. (DOC 216 kb) Additional file 2: SPIRIT checklist. A table specifying where any SPIRIT item has been addressed in the protocol manuscript. (DOC 121 kb)
